# In Silico Molecular Docking and In Vivo Validation with *Caenorhabditis elegans* to Discover Molecular Initiating Events in Adverse Outcome Pathway Framework: Case Study on Endocrine-Disrupting Chemicals with Estrogen and Androgen Receptors

**DOI:** 10.3390/ijms20051209

**Published:** 2019-03-10

**Authors:** Jaeseong Jeong, Hunbeen Kim, Jinhee Choi

**Affiliations:** School of Environmental Engineering, University of Seoul, 163 Seoulsiripdae-ro, Dongdaemun-gu, Seoul 02504, Korea; erphios@naver.com (J.J.); hunbin10000@naver.com (H.K.)

**Keywords:** molecular initiating event, endocrine-disrupting chemicals, molecular docking, reproductive toxicity, *Caenorhabditis elegans*, Tox21

## Abstract

Molecular docking is used to analyze structural complexes of a target with its ligand for understanding the chemical and structural basis of target specificity. This method has the potential to be applied for discovering molecular initiating events (MIEs) in the Adverse Outcome Pathway framework. In this study, we aimed to develop in silico–in vivo combined approach as a tool for identifying potential MIEs. We used environmental chemicals from Tox21 database to identify potential endocrine-disrupting chemicals (EDCs) through molecular docking simulation, using estrogen receptor (ER), androgen receptor (AR) and their homology models in the nematode *Caenorhabditis elegans* (NHR-14 and NHR-69, respectively). In vivo validation was conducted on the selected EDCs with *C. elegans* reproductive toxicity assay using wildtype N2, *nhr-14*, and *nhr-69* loss-of-function mutant strains. The chemicals showed high binding affinity to tested receptors and showed the high in vivo reproductive toxicity, and this was further confirmed using the mutant strains. The present study demonstrates that the binding affinity from the molecular docking potentially correlates with in vivo toxicity. These results prove that our in silico–in vivo combined approach has the potential to be applied for identifying MIEs. This study also suggests the potential of *C. elegans* as useful in the in vivo model for validating the in silico approach.

## 1. Introduction

The use of in silico approaches in chemical toxicity tests is anticipated to increase in a variety of applications and to address a number of regulatory challenges [1,2]. In silico approaches can be used to support read-across, prioritization, and screening. Among various in silico approaches, molecular docking, where toxicity is predicted based on the ligand-receptor complex structure and binding affinity, is a promising tool for chemical toxicity screening [3,4]. Molecular docking is a computational ligand-target docking approach that has been used to analyze structural complexes of a target with its ligand to understand the chemical and structural basis of a ligand’s target specificity. Molecular docking has the potential to be applied for discovering molecular initiating events (MIEs) in the Adverse Outcome Pathway (AOP) framework [5]. The conceptual AOP framework has been presented as a logical sequence of events or processes within biological systems that can be used to understand adverse effects and refine current risk assessment practices [6]. Within the AOP framework, the MIE is defined as the first point of chemical-biological interaction within an organism that starts the AOP.

It is well-established that endocrine-disrupting chemicals (EDCs) interfere with hormonal signaling, which is mediated by nuclear receptor (NR) family proteins [7] such as estrogen receptor alpha (ERα) and androgen receptor (AR). Direct binding to NRs is one of the main mechanisms by which EDCs can affect the endocrine system [8]. The interaction between a receptor and its ligand is one of the first reactions in the toxicity pathway of chemicals in the AOP framework concept [9]. There is strong evidence that ER and AR can be the MIE of the AOP framework, such as androgen receptor agonism leading to reproductive dysfunction (https://aopwiki.org/aops/23), androgen receptor activation leading to hepatocellular adenomas and carcinomas (https://aopwiki.org/aops/117) and estrogen receptor agonism leading to reproductive dysfunction (https://aopwiki.org/aops/30).

In vivo validation of data driven by in silico molecular docking would increase the potential of molecular docking as an alternative approach for animal toxicity testing. Among various in vivo models, *Caenorhabditis elegans*, a small nematode that can be maintained at low cost and handled using standard in vitro techniques, is a powerful surrogate model for mammalian toxicity tests [10,11,12,13]. Indeed, toxicity ranking screening in *C. elegans* has been shown to predict median lethal dose (LD50) ranking in rats and mice [14,15,16,17,18,19]. Moreover, many modes of action of toxicity have been reported to be conserved between *C. elegans* and mammals [11,12,20,21,22]. These consistent correlations suggest *C. elegans* assays may be valuable in early safety testing as one component in tiered or integrated toxicity testing strategies.

In this context, to develop a *C. elegans* based in silico-in vivo integrated test, we conducted in silico molecular docking analysis on potential EDCs with *C. elegans* homology models of human ERα and AR. The lists of ligands were downloaded from PubChem Tox21 summary data on agonists of ERα and AR. Molecular docking analysis was conducted using two endogenous hormones and 33 environmental chemicals. Finally, in vivo validation using *C. elegans* wildtype and mutant strains was conducted to test the feasibility of ligand-receptor binding affinity for screening for and/or predicting toxicity for discovering the MIEs in an AOP framework.

## 2. Results and Discussion

### 2.1. Preparation of Ligands from Tox21 Assays

The ligands were prepared from PubChem (https://pubchem.ncbi.nlm.nih.gov) Tox21 assays summary (PubChem ID 743077 for ERα agonists, ID 743078 for ERα antagonists, ID 743053 for AR agonists, and ID 743063 for AR antagonists). These assays are cell-based assays that use HEK293T, a human kidney cell line measuring gene activity via a fluorescent protein reporter gene to screen the Tox21 10K compound library (10,486 chemicals). As the results of the Tox21 assay, chemicals are divided into active and inactive, only the active chemicals can bind to the ligand-binding domain (LBD) of the protein, resulting in expression of the fluorescent reporter gene. Since they can bind to ERα or AR, the active chemicals can be considered as potential EDCs. The sets of active chemicals consisted of 1348 chemicals for ERα and 1451 chemicals for AR. Among them, 33 active chemicals included in both ERα and AR assays (2 endogenous hormones and 31 environmental chemicals) were selected for docking studies. In addition to the active chemicals, NP and DEHP were selected as well-known EDCs but not on the active list. A full list of the ligands was provided in Table S1.

### 2.2. Homology Modeling of C. elegans Receptors

**Figure 1:**
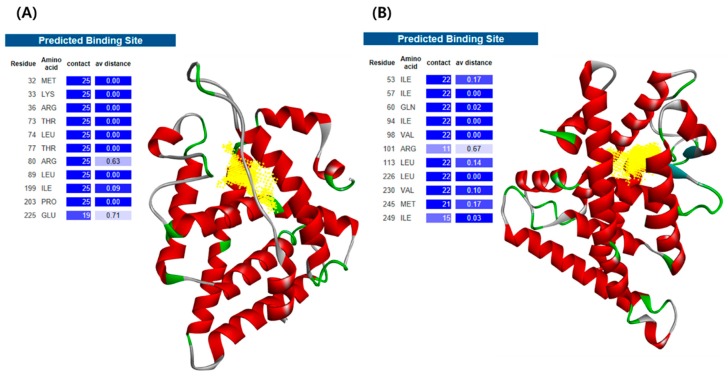
The 3D structure of *C. elegans* receptors, (**A**) NHR-14 and (**B**) NHR-69 predicted by Protein Homology/analogY Recognition Engine V2.0 (PHYRE2) server, and their protein ligand binding residues predicted by 3DLigandSite. The homology model was colored according to the secondary structure, and the ligand binding site was displayed in yellow.

As a first step to assess the toxicity of environmental chemicals by integrating in silico and in vivo methods in *C. elegans*, we established homology models of *C. elegans* receptors. In designing *C. elegans* receptors, target receptors were selected that met the following three criteria: (i) receptors homologous to human ERα and AR; (ii) receptors that are experimentally validated to behave like specific human receptors; and (iii) receptors with known sequential information. As a result, for *C. elegans* receptors homology modeling, NHR-14 and NHR-69 was selected as it was reported to be orthologous to human ERα and AR, respectively, by sequence similarity and confirming binding with natural ligands [23,24]. With the LBD sequence information of *C. elegans* receptors from UniProt, three-dimensional (3D) structures of the receptors were built using PHYRE2 (Figure 1) [25]. The ligand-binding site of each receptor was predicted by the 3DLigandSite (Figure 1) [26].

The NHR-14 and NHR-69 obtained a ProSA-web Z-score of −5.73 and −7.3, respectively, which is well within the range of values observed for all experimentally determined protein chains in PBD (Figure S1). Further, the Ramachandran plot revealed that about 97.3% (NHR-14) and 99.2% (NHR-69) residues are in the favored and allowed region, whereas 2.7% and 0.8% residues are in outlier region, respectively (Figure S2). These results indicate that the generated model structures were good for further docking studies.

### 2.3. Docking Simulation with Human Receptor

Molecular docking simulation was carried out with the 35 ligands to the binding site of the two human receptors (ERα and AR) using AutoDock Vina v1.1 docking software (Table 1). For each ligand, out of the many docking poses, only those that possessed the highest docking score were chosen. The two endogenous hormone ligands, 17β-estradiol, the main estrogen, and testosterone, the main androgen, showed the same binding affinity of −10.5 kcal/mol with ERα and AR, respectively. When we interpreted these predicted binding affinity results, the large absolute value of the energy means the corresponding ligand-receptor reaction has a great affinity as this data represents the free energy of binding in AutoDock Vina v1.1 docking software [27]. Therefore, considering its role in each hormone system, binding results with endogenous hormone ligands agree well with the expected values. Benzo[k]fluoranthene, benzo[a]pyrene, 7-methylbenzo[a]pyrene and 9,10-dihydrobenzo[a]pyren-7(8H)-one were in the top five environmental chemical ligands in both receptors having a binding affinity of −9.8 kcal/mol or below (Table 1). These chemicals are polycyclic aromatic hydrocarbons (PAHs), which are well known as EDCs [28,29]. The binding ability of these chemicals to ERα and AR was similar to that of endogenous hormone, and these results are consistent with previous studies that reported relationships between EDCs and nuclear receptors [30,31,32]. In silico approach to predict potential binding tendency between EDCs and the receptors was also performed by Kolšek et al. as a web platform ‘Endocrine Disruptome’ [33] and by Grignard et al. [34].

### 2.4. Docking Simulation with C. elegans Receptors

To verify whether the binding tendencies of human receptors are conserved in *C. elegans*, molecular docking simulation was carried out with the *C. elegans* models (NHR-14 and NHR-69) using AutoDock Vina (Table 1). Again, for each ligand, out of the many docking poses, only those that possessed the highest docking scores were chosen. Interestingly, the endogenous hormone ligands, 17β-estradiol and testosterone showed the same binding affinity of −8.3 kcal/mol on NHR-14 and NHR-69 respectively as human results, although binding is weaker than on human receptors (−10.5 kcal/mol). Benzo[k]fluoranthene, benzo[a]pyrene, 7-methylbenzo[a]pyrene, benzo[b]fluoranthene and 9,10-dihydrobenzo[a]pyren-7(8H)-one were the top five environmental chemical ligands in both receptors (Table 1). These results appear to be very similar to those in human receptors, suggesting that our *C. elegans* models can surrogate the human receptors well. Although the binding affinity of NHR-14 was generally higher than the binding affinity of NHR-69, the rank-order of binding affinity is more important in the molecular docking study than the binding affinity itself, because the scoring function is different for each docking software [35,36].

**Figure 2:**
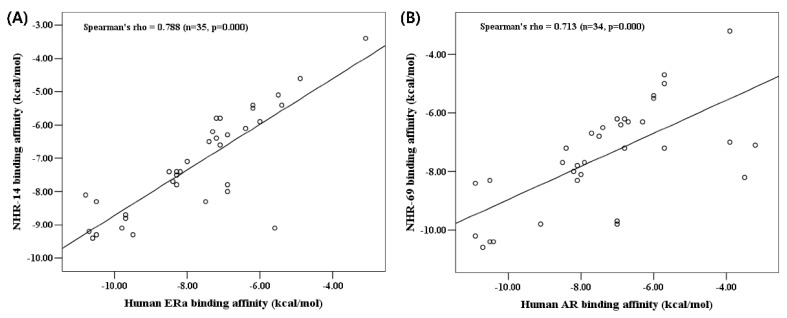
Correlation analysis of binding affinity. Correlation plot between (**A**) human estrogen receptor alpha and its homology model of *C. elegans*, NHR-14 and (**B**) human androgen receptor and its homology model of *C. elegans*, NHR-69. Correlation analysis was performed by Spearman’s rank-order correlation.

As a result of the correlation analysis between the binding affinity of human receptors and their *C. elegans* homology models, the Spearman’s rank-order correlation coefficient between ERα and NHR-14 was 0.788, and AR and NHR-69 was 0.713 (Figure 2). Therefore, it was confirmed that there is a high correlation between the human receptors and our homology model for environmental chemicals. This result suggests that the homology model was suitable and that our candidate *C. elegans* receptors have immense potential as tools to examine the toxicity pathway induced by environmental chemicals.

### 2.5. Experimental Validation on NHR-14 and NHR-69

**Figure 3:**
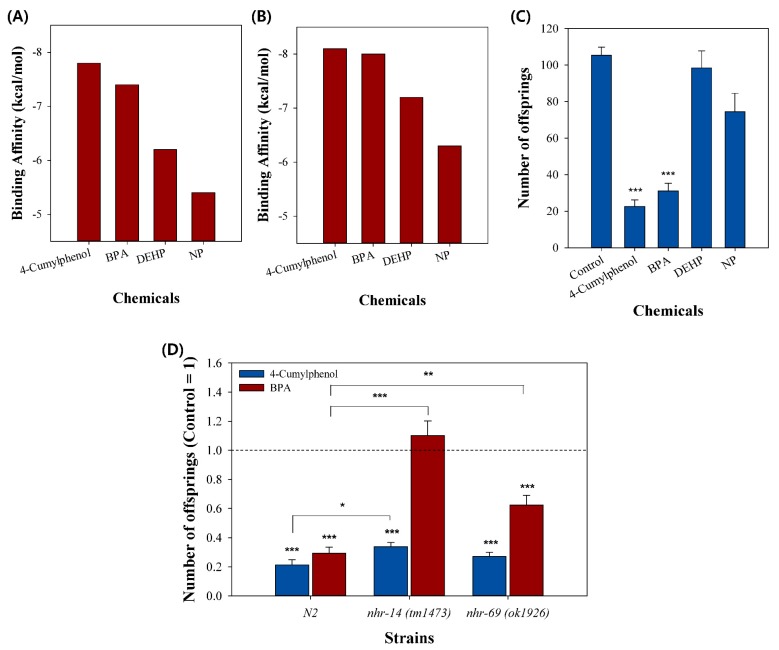
Experimental validation of selected endocrine-disrupting chemicals (EDCs). Binding affinity of 4-cumylphenol, bisphenol A (BPA), bis(2-ethylhexyl)phthalate (DEHP), and 4-nonylphenol (NP) to (**A**) NHR-14 and (**B**) NHR-69. (**C**) Reproductive toxicity of 4-cumylphenol, BPA, DEHP, and NP in wildtype *C. elegans*. (**D**) Reproductive toxicity of 4-cumylphenol and BPA on wildtype N2, *nhr-14* (*tm1473*), and *nhr-69* (*ok1926*) mutants. * *p* < 0.05, ** *p* < 0.01, *** *p* < 0.001.

For in vivo validation of the in silico molecular docking simulation, we selected four chemicals, 4-cumylphenol, bisphenol A (BPA), 4-nonylphenol (NP), and bis(2-ethylhexyl)phthalate (DEHP). Several studies have reported that these compounds interfere with various hormone receptors by inhibiting their normal interactions with natural hormone ligands, resulting in reproductive toxicity [32,37,38,39,40,41,42]. In molecular docking, 4-cumylphenol showed the highest binding affinity in both NHR-14 and NHR-69, followed by BPA, DEHP and NP (Figure 3A,B).

Validation with the *C. elegans* reproduction assay revealed that 4-cumylphenol and BPA exposure caused significant toxicity to *C. elegans*, which was not observed upon DEHP and NP exposure (Figure 3C). As a result of the significant toxicity when exposed to chemicals with high binding affinity, 4-cumylphenol and BPA, the toxicity of the two chemicals seems to be caused by the binding to NHR-14 and NHR-69. This is consistent with previous research suggesting that 4-cumylphenol and BPA have high estrogenic potential [43,44]. The subsequent mutant assay revealed that reproductive toxicity by 4-cumylphenol was slightly rescued in *nhr-14*, and BPA was significantly rescued in both *nhr-14* and *nhr-69* loss-of-function mutant strains, suggesting their functional role in reproduction (Figure 3D).

Collectively with the model organism *C. elegans*, ligand docking simulation with selected environmental chemicals results were similar to the human ones, reproductive toxicity was revealed, and through mutant testing, NHR-14 and NHR-69 were verified as influencing factors in the expression of toxicity. Based on these results, we found that NHR-14 and NHR-69 are related to reproductive toxicity of environmental chemicals, and molecular binding to these receptors may be MIEs for this adverse outcome (AO). Collectively, our results demonstrate that the rank of binding affinity from molecular docking was well correlated with that of in vivo toxicity, which prove the in silico-in vivo combined approach has a potential to be applied to toxicity screening, such as, identification of the MIEs in the AOP framework.

## 3. Materials and Methods

### 3.1. Study Design

**Figure 4:**
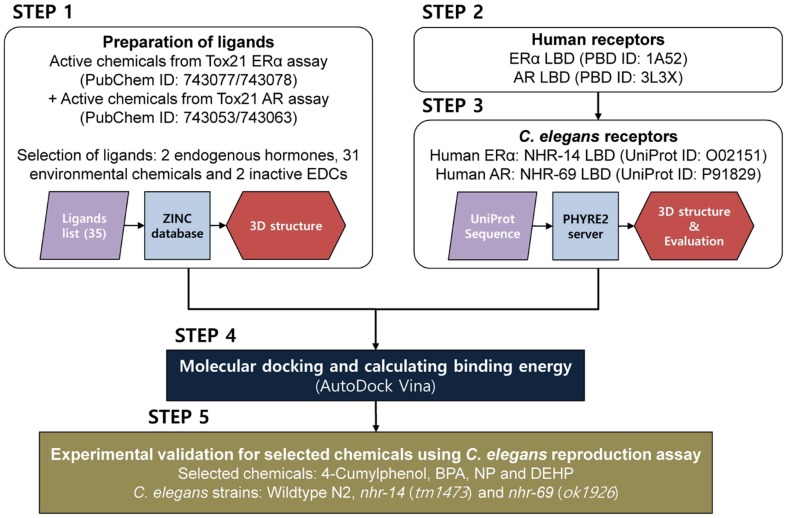
Overall study design and workflow.

Figure 4 depicts the overall study design and workflow.

1. Step 1: Active chemicals from Tox21 ERα agonists/antagonists (PubChem ID: 743077/743078) and AR agonists/antagonists (PubChem ID: 743053/743063) assays were used as ligands for docking analysis.
2. Step 2: Human ERα and AR LBDs were prepared from the Protein Data Bank (PDB).
3. Step 3: The LBD sequences of *C. elegans* nuclear hormone receptor (NHR) known to homologous to human ERα and AR were collected from UniProt. 3D structures of each protein were built using homology modeling by PHYRE2 server, and the binding grids of each structure were defined.
4. Step 4: The optimized ligands were then docked into the binding grid of each 3D human ERα and AR and *C. elegans* NHR structures using AutoDock Vina. The order of the calculated binding free energy was compared between human and *C. elegans* receptors.
5. Step 5: Experimental validation was conducted using *C. elegans* reproduction assay on the selected chemicals.

### 3.2. Preparation of Ligands

We used active chemicals of ERα and AR for docking simulation of each human and *C. elegans* receptor. The lists of the active chemicals were obtained from PubChem (https://pubchem.ncbi.nlm.nih.gov) Tox21 assays summary (PubChem ID 743077 for ERα agonists, ID 743078 for ERα antagonists, ID 743053 for AR agonists, and ID 743063 for AR antagonists). Among them, the chemicals that have no structural information were excluded, and the 2 endogenous hormones and 31 environmental chemicals that showed “active” in four assays (agonists or antagonists to both receptors) were selected for the docking simulation. Additionally, well-reported EDCs, NP and DEHP were also selected as a target chemical. The 3D structures of all ligands were collected in MOL2 format from the ZINC database [45]. These files could not be directly used for docking simulation, thus they were converted it into PDBQT format using AutoDockTools v1.5.6 [46,47].

### 3.3. Preparation of Human Receptors

Human ERα and AR were used as target proteins for screening EDCs. The coordinates of the X-ray crystal structures of LBDs of the receptors were retrieved from the Protein Data Bank (PDB) [48]. Structures 1A52 and 3L3X were selected for ERα and AR, respectively. The structures were edited to remove ligands and heteroatoms (HETATM) using Discovery Studio Visualizer v4.5 (BIOVIA, San Diego, CA, USA).

### 3.4. Homology Modeling of C. elegans Receptors

The LBD amino acid sequences of *C. elegans* NHR-14 and NHR-69 were downloaded from UniProt Database (https://www.uniprot.org) with accession number O02151 and P91829, respectively (Figure S3). Using the obtained sequences, 3D protein structure models were predicted by the Protein Homology/analogY Recognition Engine V2.0 (PHYRE2) server [25]. PHYRE2 ranks homologous proteins as a template; the Alignment Coverage PDB entry codes of the best scored template for NHR-14 receptor was 1XPC, and NHR-69 receptor was 1HG4. The ligand-binding sites of each protein were predicted by the 3DLigandSite server (Figure 1) [26]. 3Drefine web server was used for protein structure refinement of the models. The 3Drefine refinement utilizes iterative optimization of hydrogen bonding network combined with atomic-level energy minimization [49]. ProSA-web and RAMPAGE server were used for model evaluation. ProSA-web was used to calculate the Z-score indicating overall model quality of the 3D structure [50]. RAMPAGE server was used to generate a Ramachandran 2D contour plot to predict the stereochemical quality of the 3D structures [51].

### 3.5. Docking Simulations

We used AutoDock Vina v1.1 [27] programs to investigate the binding of ligands to receptors. Required input files for AutoDock Vina were prepared using AutoDockTools v1.5.6 (The Scripps Research Institute, La Jolla, CA, USA). Preparation of files involved changing atom type, removing water molecules, and adding polar hydrogen atoms and Gasteiger charges. The grid box size was kept as 22, 22, and 22 for X, Y, and Z, and the grid points spacing was 1 Å. The structure files were saved in PDBQT format. Molecular docking analysis was performed using AutoDock Vina v1.1 (The Scripps Research Institute). The exhaustiveness was set to 128 and the maximum number of simultaneous threads was set to 2. The results with best conformation and energetic were selected for analysis. Discovery Studio Visualizer v4.5 (BIOVIA, San Diego, CA, USA) was used for visualization and analysis of the protein-ligand complexes.

### 3.6. C. elegans Reproduction Assay

For reproduction assay, 4-cumylphenol, BPA, NP, and DEHP were purchased from Sigma-Aldrich (St. Louis, MO, USA). *C. elegans* were grown in petri dishes on nematode growth medium (NGM) and fed *OP50* strain *Escherichia coli* according to a standard protocol [52]. Worms were incubated at 20 °C, and age-synchronized young adults (3 days after the age-synchronizing procedure) were used in 72-h reproduction assay. Wildtype N2 and *nhr-69* (*ok1926*) were provided by the Caenorhabditis Genetics Center (www.CGC.org) at the University of Minnesota (Minneapolis, MN, USA). The *nhr-14* (*tm1473*) was provided by the National Bioresource Project for the nematode (S. Mitani, Department of Physiology, Tokyo Women’s Medical University School of Medicine, Tokyo, Japan). The reproduction test was conducted on wildtype N2 and mutant strains by measuring the number of offspring from one young-adult worm after 72 h of exposure using complex object parametric analysis and sorting (COPAS)-SELECT. *C. elegans* strains were exposed to each chemical at 0.5 mM.

### 3.7. Statistical Analysis

The significance of differences between treatments was determined using one-way analysis of variance (ANOVA) followed by a post-hoc test (Tukey, *p* < 0.05) and correlation analysis was performed by Spearman’s rank-order correlation test. All statistical analyses were performed in SPSS 13.0 (SPSS Inc., Chicago, IL, USA). Graphs were prepared in SigmaPlot (Version 12.0, Systat Software Inc., San Jose, CA, USA).

## 4. Conclusions

In this study, to develop a *C. elegans*-based in silico-in vivo integrated test, in silico molecular docking simulations of *C. elegans* NHRs and the 33 environmental chemicals were conducted and compared to the results of human receptors and in vivo reproductive toxicity test. The molecular docking results of *C. elegans* NHR-14 (human ERα homologous) and NHR-69 (human AR homologous) were highly correlated with those of human receptors, and the top five ligands as rank-ordered by binding affinity were very similar. In the reproductive analysis, among the four selected EDCs, 4-cumylphenol showed the highest binding affinity, and showed the highest in vivo reproductive toxicity. We found NHR-14 and NHR-69 are related with reproductive toxicity of environmental chemicals, and comparing molecular docking and reproductive toxicity results indicate that the binding affinity from the molecular docking is potentially correlated with reproductive toxicity.

We proposed potential EDCs using Tox21 assay and *C. elegans*-based in silico-in vivo test. The use of the Tox21 high throughput screening experimental data integrated with the in silico-in vivo test using *C. elegans* has the advantage of quickly identifying the endocrine-disrupting potential of large quantities of chemicals. In addition, the results of the correlation analysis of the human-*C. elegans* binding affinity indicate that *C. elegans* have a potential to be used as an alternative model for EDCs screening of environmental chemicals.

We identified the feasibility of applying molecular docking simulations to screen for MIEs of AOs. In other words, a high level of ligand-receptor binding could be an important signal that a chemical exerts a potential risk. These results showed that the in silico molecular docking model can be a potential tool for screening and/or predicting toxicity pathway for discovering the MIE in an AOP framework.

## Figures and Tables

**Table 1:** Calculated binding affinity of the ligands on human estrogen receptor alpha (ERα) and androgen receptor (AR), and *C. elegans* NHR-14 and NHR-69.

No.	Chemical		Estrogen Receptor Alpha		Androgen Receptor	
	Name	CAS No.	ERα (kcal/mol)	NHR-14 (kcal/mol)	AR (kcal/mol)	NHR-69 (kcal/mol)
1	17beta-Estradiol	50-28-2	−10.5	−8.3	−10.9	−8.4
2	Testosterone	58-22-0	−10.8	−8.1	−10.5	−8.3
3	1,2-Benzenedicarboxaldehyde	643-79-8	−5.4	−5.4	−5.7	−5.0
4	1,3-Diiminobenz[f]isoindoline	65558-69-2	−8.3	−7.5	−8.4	−7.2
5	1,6-Hexanediol diacrylate	13048-33-4	−5.5	−5.1	−6.0	−5.4
6	10-Chloro-9-anthraldehyde	10527-16-9	−8.0	−7.1	−7.9	−7.7
7	2,2′-Methylenebis(4-methyl-6-tert-butylphenol)	119-47-1	−6.9	−8.0	−3.5	−8.2
8	2,4-Bis(1-methyl-1-phenylethyl)phenol	2772-45-4	−9.7 *	−8.7	−7.0	−9.8 *
9	2-Aminoanthracene	613-13-8	−8.5	−7.4	−8.5	−7.7
10	4,4′-Thiobis(6-tert-butyl-m-cresol)	96-69-5	−6.9	−7.8	−3.2	−7.1
11	4,6-Di-tert-butyl-m-cresol	497-39-2	−6.9	−6.3	−6.8	−7.2
12	4-Cumylphenol	599-64-4	−8.3	−7.8	−8.0	−8.1
13	4-Nitrosodiphenylamine	156-10-5	−7.1	−6.6	−7.5	−6.8
14	4-Nonylphenol	104-40-5	−6.2	−5.4	−6.3	−6.3
15	7-(Dimethylamino)-4-methylcoumarin	87-01-4	−7.2	−6.4	−7.4	−6.5
16	7-Diethylamino-4-methylcoumarin	91-44-1	−7.4	−6.5	−7.7	−6.7
17	7-Methylbenzo[a]pyrene	63041-77-0	−10.6 *	−9.4 *	−10.5 *	−10.4 *
18	9,10-Dihydrobenzo[a]pyren-7(8H)-one	3331-46-2	−10.5 *	−9.3 *	−10.4 *	−10.4 *
19	9-Bromoanthracene	1564-64-3	−8.2	−7.4	−8.1	−7.8
20	9-Cyanoanthracene	1210-12-4	−8.4	−7.7	−8.1	−8.3
21	alpha-Terthiophene	1081-34-1	−6.4	−6.1	−6.8	−6.2
22	Benzo[a]pyrene	50-32-8	−10.7 *	−9.2 *	−10.7 *	−10.6 *
23	Benzo[b]fluoranthene	205-99-2	−9.5	−9.3 *	−9.1 *	−9.8 *
24	Benzo[e]pyrene	192-97-2	−9.7 *	−8.8	−7.0	−9.7
25	Benzo[k]fluoranthene	207-08-9	−9.8 *	−9.1 *	−10.9 *	−10.2 *
26	Bis(2-Ethylhexyl)phthalate (DEHP)	117-81-7	−7.3	−6.2	−5.7	−7.2
27	Bisphenol A	80-05-07	−8.3	−7.4	−8.2	−8.0
28	Chlorothalonil	1897-45-6	−6.0	−5.9	−6.7	−6.3
29	Crystal Violet lactone	1552-42-7	−5.6	−9.1 *	NA	−7.3
30	Dodecyl gallate	1166-52-5	−7.2	−5.8	−6.9	−6.4
31	Ethylene acrylate	2274-11-5	−4.9	−4.6	−5.7	−4.7
32	Fluazinam	79622-59-6	−7.5	−8.3	−3.9	−7.0
33	Octyl gallate	1034-01-1	−7.1	−5.8	−7.0	−6.2
34	Tribromoacetaldehyde	115-17-3	−3.1	−3.4	−3.9	−3.2
35	Trimethylolpropane triacrylate	15625-89-5	−6.2	−5.5	−6.0	−5.5

* Top 5 environmental chemicals for each receptor.

## Supplementary Materials

Supplementary materials can be found at https://www.mdpi.com/1422-0067/20/5/1209/s1.

## Abbreviations

AOP	Adverse Outcome Pathway
AR	Androgen Receptor
BPA	Bisphenol A
DEHP	Bis(2-ethylhexyl)phthalate
EDCs	Endocrine-disrupting Chemicals
ER	Estrogen Receptor
LBD	Ligand Binding Domain
MIE	Molecular Initiating Event
NHR	Nuclear Hormone Receptor
NP	4-Nonylphenol
