# Supplementary material for: In Silico Molecular Docking and In Vivo Validation with Caenorhabditis elegans to Discover Molecular Initiating Events in Adverse Outcome Pathway Framework: Case Study on Endocrine-Disrupting Chemicals with Estrogen and Androgen Receptors
Source: Int J Mol Sci. 2019 Mar 10;20(5):1209. doi: 10.3390/ijms20051209 (PMC6429066; doi:10.3390/ijms20051209)
Supplement: Supplementary file 1 [file ijms-20-01209-s001.pdf]

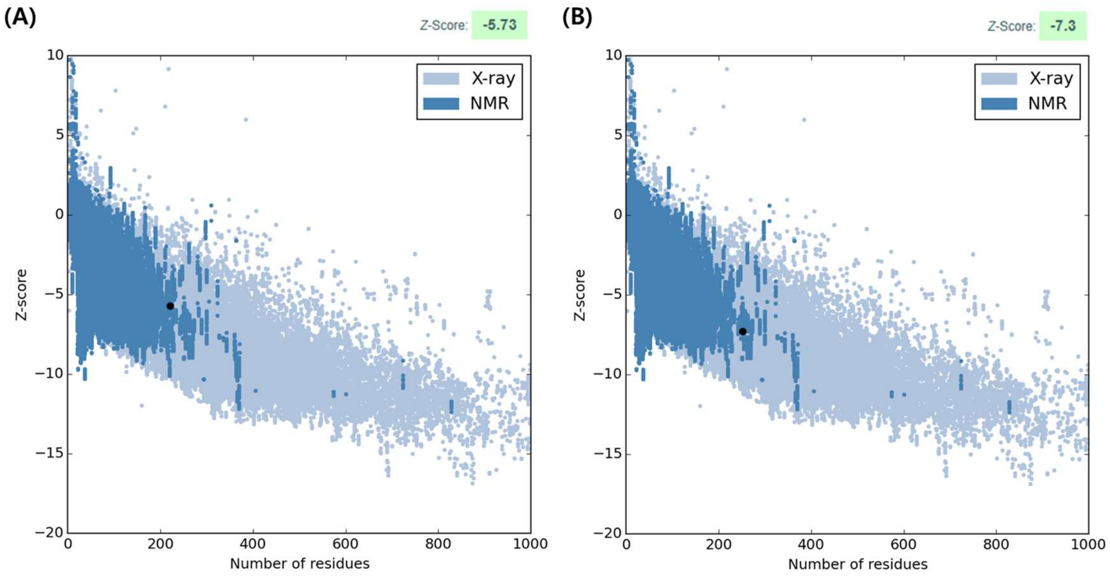

Figure S1. Overall model quality of *C. elegans* receptors, (**A**) NHR-14 and (**B**) NHR-69 analyzed by ProSA-web. The Z-scores (represented by a black dot) were represented in the range of the Z-score for all proteins in Protein Data Bank of which structures are determined by X-ray crystallography (light blue region) and nuclear magnetic resonance (NMR; dark blue region).

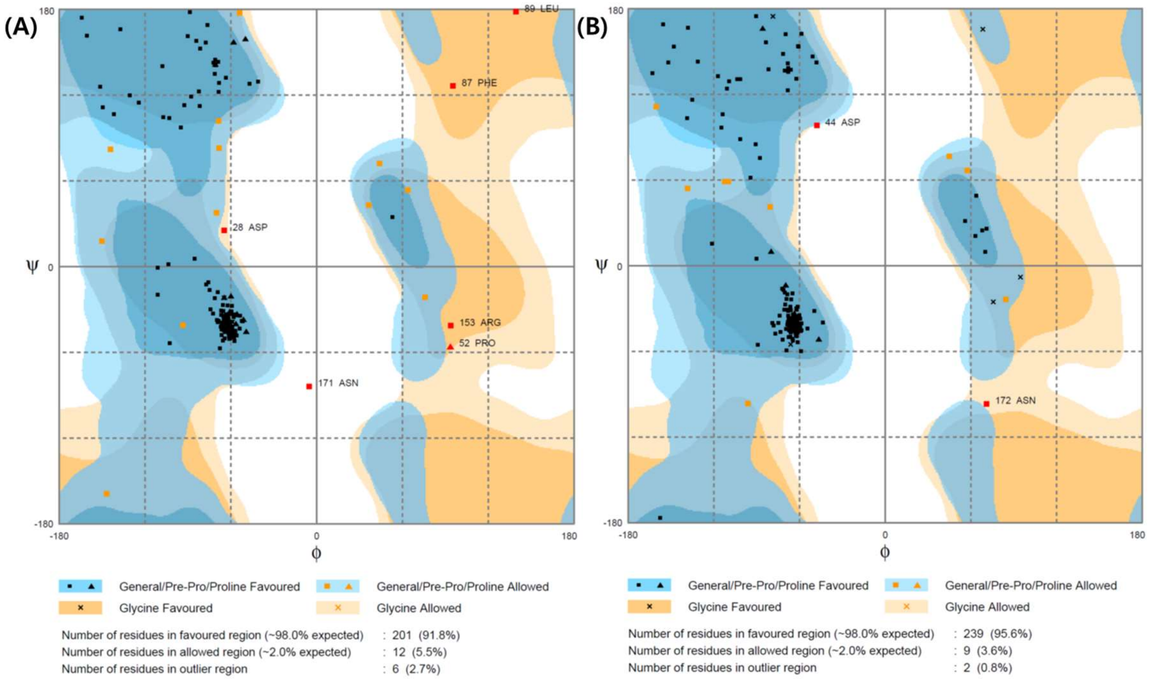

Figure 2. A 2D-Ramachandran Plot of *C. elegans* receptors, (**A**) NHR-14 and (**B**) NHR-69 analyzed by RAMPAGE server.

(A)

```
        10         20         30         40         50
MDFLISTSLS ESTSTSADFC VVCGDKAIGK HYGAVACNGC KGFFRRSVWQ
        60         70         80         90        100
NLQYTCRFNK QCNIDKDHRN ACRYCRFQKC LADGMKPEAI QNERDRIGST
       110        120        130        140        150
KRRKRSGANS ENNSDSEGTP SPKIEVMGNS VSRKLIEMLL DIEHRLASNQ
       160        170        180        190        200
SMNALLRDES EMKNSRQRAV NYLIGWTNML HPLPEVPLAD KVLLLKKFSS
       210        220        230        240        250
AFTLLGTLQR SMALPHFVLP NDQVLSISAS HPPELFEALT RIIDELLTPL
       260        270        280        290        300
RRLRTDHAEF SCLKALLLLN PDVVGISNNT RERIREARDA LLKTLFAYMS
       310        320        330        340        350
NTQNSIDASL RVSSLLMIIP SLISVSSSIM EFPALSDLFG LGDVIKRDTI
       360        370        380        390        400
SPKIETPPLE MKPMMPKIAQ PPVTSAPTVP TNIMMNKDLI SQIMNNPQLF
       410        420        430
PLLPMPQTAS PPMSFMGQSE FGCHLQSMPV KVILS
```

(B)

```
        10         20         30         40         50
MVEEICHICN DKSTGKHYGA ISCDGCKGFF RRSIRKRYHY QCRFEQNCDV
        60         70         80         90        100
TKNKRNACRA CRLQKCVKAG MKSNAIQNER DAIGKRKKTT GAEKEDLIDQ
       110        120        130        140        150
LVAAETLCQQ LRSSVIKNTS SLAPYDCGKV KWNYEDARAA TLDDIGKSIH
       160        170        180        190        200
QQLVLFIEWA KSLPQFSFLA QADQAALLKG GAASIIVLGV AYRSICLTVE
       210        220        230        240        250
NTICLANDTL LPKEHATQVG DINCVVGRII DEIVNPMRRL NMDLIEYVAL
       260        270        280        290        300
KAILFFNPVV REINDQSPVE NARYAFLRSL QRRCTDKALE NMEDESMDCR
       310        320        330        340        350
SGKLLLLLPS LQAIAQQLVE DVQLARLFGL VNVDSLMEEL ILNDMKPSDP
       360        370
QILQTSLASP VNSSVKAEVE LEE
```

Figure S3. Sequences information of *C. elegans* receptors, (**A**) NHR-14 and (**B**) NHR-69 from UniProt Database with accession number O02151 and P91829, respectively. The ligand-binding domain is highlighted in yellow.

Table 1. A full list of the ligands.

No.	Chemical			
	Name	CAS No.	SMILES	Category
1	17beta-Estradiol	50-28-2	C[C@]12CC[C@H]3[C@@H](CCC4=CC(O)=CC=C34)[C@@H]1CC[C@@H]2O	Endogenous hormone
2	Testosterone	58-22-0	C[C@]12CC[C@H]3[C@@H](CCC4=CC(=O)CC[C@]34C)[C@@H]1CC[C@@H]2O	Endogenous hormone
3	1,2-Benzenedicarboxaldehyde	643-79-8	O=CC1=CC=CC=C1C=O	Environmental chemical
4	1,3-Diiminobenz[f]isoindoline	65558-69-2	N=C1NC(=N)C2=C1C=C1C=CC=CC1=C2	Environmental chemical
5	1,6-Hexanediol diacrylate	13048-33-4	C=CC(=O)OCCCCCCOC(=O)C=C	Environmental chemical
6	10-Chloro-9-anthraldehyde	10527-16-9	ClC1=C2C=CC=CC2=C(C=O)C2=CC=CC=C12	Environmental chemical
7	2,2'-Methylenebis(4-methyl-6-tert-butylphenol)	119-47-1	CC1=CC(CC2=CC(C)=CC(=C2O)C(C)(C)C)=C(O)C(=C1)C(C)(C)C	Environmental chemical
8	2,4-Bis(1-methyl-1-phenylethyl)phenol	2772-45-4	CC(C)(C1=CC=CC=C1)C1=CC(=C(O)C=C1)C(C)(C)C1=CC=CC=C1	Environmental chemical
9	2-Aminoanthracene	613-13-8	NC1=CC=C2C=C3C=CC=CC3=CC2=C1	Environmental chemical
10	4,4'-Thiobis(6-tert-butyl-m-cresol)	96-69-5	CC1=CC(O)=C(C=C1SC1=C(C)C=C(O)C(=C1)C(C)(C)C)C(C)(C)C	Environmental chemical
11	4,6-Di-tert-butyl-m-cresol	497-39-2	CC1=C(C=C(C(O)=C1)C(C)(C)C)C(C)(C)C	Environmental chemical
12	4-Cumylphenol	599-64-4	CC(C)(C1=CC=CC=C1)C1=CC=C(O)C=C1	Environmental chemical
13	4-Nitrosodiphenylamine	156-10-5	O=NC1=CC=C(NC2=CC=CC=C2)C=C1	Environmental chemical
14	4-Nonylphenol	104-40-5	CCCCCCCCCC1=CC=C(O)C=C1	Environmental chemical
15	7-(Dimethylamino)-4-methylcoumarin	87-01-4	CN(C)C1=CC2=C(C=C1)C(C)=CC(=O)O2	Environmental chemical
16	7-Diethylamino-4-methylcoumarin	91-44-1	CCN(CC)C1=CC=C2C(C)=CC(=O)OC2=C1	Environmental chemical
17	7-Methylbenzo[a]pyrene	63041-77-0	CC1=C2C=C3C=CC4=C5C(C=CC(C2=CC=C1)=C35)=CC=C4	Environmental chemical
18	9,10-Dihydrobenzo[a]pyren-7(8H)-one	3331-46-2	O=C1CCCC2=C1C=C1C=CC3=C4C(C=CC2=C14)=CC=C3	Environmental chemical
19	9-Bromoanthracene	1564-64-3	BrC1=C2C=CC=CC2=CC2=CC=CC=C12	Environmental chemical
20	9-Cyanoanthracene	1210-12-4	N#CC1=C2C=CC=CC2=CC2=C1C=CC=C2	Environmental chemical
21	alpha-Terthiophene	1081-34-1	S1C=CC=C1C1=CC=C(S1)C1=CC=CS1	Environmental chemical
22	Benzo[a]pyrene	50-32-8	C1=CC2=CC3=CC=C4C=CC=C5C=CC(=C2C=C1)C3=C45	Environmental chemical
23	Benzo[b]fluoranthene	205-99-2	C1=CC2=C(C=C1)C1=CC3=C(C=CC=C3)C3=C1C2=CC=C3	Environmental chemical
24	Benzo[e]pyrene	192-97-2	C1=CC2=C3C=CC=C4C=CC5=CC=CC(=C2C=C1)C5=C34	Environmental chemical
25	Benzo[k]fluoranthene	207-08-9	C1=CC2=CC3=C(C=C2C=C1)C1=C2C3=CC=CC2=CC=C1	Environmental chemical
26	Bis(2-Ethylhexyl)phthalate (DEHP)	117-81-7	CCCC[C@H](CC)COC(=O)c1ccccc1C(=O)OC[C@H](CC)CCCC	Environmental chemical
27	Bisphenol A	80-05-07	CC(C)(C1=CC=C(O)C=C1)C1=CC=C(O)C=C1	Environmental chemical
28	Chlorothalonil	1897-45-6	ClC1=C(Cl)C(C#N)=C(Cl)C(C#N)=C1Cl	Environmental chemical
29	Crystal Violet lactone	1552-42-7	CN(C)C1=CC=C(C=C1)C1(OC(=O)C2=C1C=CC(=C2)N(C)C)C1=CC=C(C=C1)N(C)C	Environmental chemical
30	Dodecyl gallate	1166-52-5	CCCCCCCCCCCCOC(=O)C1=CC(O)=C(O)C(O)=C1	Environmental chemical

31	Ethylene acrylate	2274-11-5	C=CC(=O)OCCOC(=O)C=C	Environmental chemical
32	Fluazinam	79622-59-6	[O-][N+](=O)C1=CC(=C(Cl)C(=C1NC1=C(Cl) C=C(C=N1)C(F)(F)F)[N+]([O-])=O)C(F)(F)F	Environmental chemical
33	Octyl gallate	1034-01-1	CCCCCCCCOC(=O)C1=CC(O)=C(O)C(O)=C1	Environmental chemical
34	Tribromoacetaldehyde	115-17-3	BrC(Br)(Br)C=O	Environmental chemical
35	Trimethylolpropane triacrylate	15625-89-5	CCC(COC(=O)C=C)(COC(=O)C=C)COC(=O) C=C	Environmental chemical
